# Brucellosis Abattoir Surveillance Using Serology and Molecular Tests Among Livestock in Free State and Limpopo Provinces, South Africa

**DOI:** 10.3390/microorganisms14061215

**Published:** 2026-05-27

**Authors:** Emmanuel Seakamela, Itumeleng Matle, Koketso Desiree Mazwi, Henriette van Heerden

**Affiliations:** 1 Bacteriology Division, Agricultural Research Council: Onderstepoort Veterinary Research, Onderstepoort, Pretoria 0110, South Africa; seakamelae@arc.agric.za; 2 Department of Veterinary Tropical Diseases, Faculty of Veterinary Science, University of Pretoria, Onderstepoort, Pretoria 0110, South Africa; 3 Department of Agriculture and Animal Health, College of Agriculture and Environmental Sciences, University of South Africa, Florida 1709, South Africa; ematlei@unisa.ac.za; 4 Department of Clinical Microbiology and Infectious Diseases, Faculty of Health Sciences, University of the Witwatersrand, Parktown, Johannesburg 2193, South Africa; koketso.mazwi@wits.ac.za

**Keywords:** brucellosis, abattoirs, cattle, sheep, Limpopo, Free State, zoonosis

## Abstract

Brucellosis remains a significant zoonotic disease globally, causing considerable economic and public health consequences, particularly in high-risk areas of South Africa (SA) with extensive animal production, including the Limpopo and Free State provinces. This study used a combination of serological, microbiological, and molecular assays to investigate the prevalence and characteristics of *Brucella* in 580 slaughtered livestock (384 cattle and 196 sheep) in Limpopo and the Free State (192 cattle and 98 sheep in each province) and to compare the diagnostic findings obtained using these assays. Tissue samples collected from each animal included liver, lung, spleen, and lymph nodes. Using the standard diagnostic rose Bengal test (RBT) and complement fixation test (CFT) in series (RBT-CFT), only 2.6% (10/384) of cattle were positive, with 2.1% (95% CI: 0.7–4.9) and 3.1% (95% CI: 1.3–6.3) in the Free State and Limpopo, respectively, while 4.7% (18/384) were positive by RBT-indirect enzyme-linked immunosorbent assay (iELISA), with 5.2% (95% CI: 2.7–9.0) and 4.2% (95% CI: 2.0–7.7) in the Free State and Limpopo, respectively. The molecular prevalence was 18.5% for *Brucella* deoxyribonucleic acid (DNA), while 97% of PCR-positive cattle tested seronegative in both provinces. Ovine samples were largely seronegative despite showing higher polymerase chain reaction (PCR) positivity (28.6%). *Brucella abortus* predominated in both species, with *B. melitensis* and occasional mixed infections also detected. Mixed infections in this study were defined as samples producing multiple species-specific bands within the same sample, consistent with the presence of more than one *Brucella* species during multiplex PCR analysis. While liver and spleen tissue provided reliable PCR detection, bacterial culture yielded a 0% isolation rate. These findings, together with reports from studies in brucellosis-endemic areas in sub-Saharan Africa, demonstrate discrepancies between traditional serological methods and molecular methods, including detection of *Brucella* DNA in seronegative animals. This may indicate that some infected animals (chronic or latent carriers) are not identified by conventional serological assays alone, particularly in endemic settings. To strengthen surveillance and enhance the detection of exposed animals, the incorporation of iELISA as a complementary tool into South Africa’s routine surveillance programmes may be beneficial, particularly when combined with regular testing of all animals.

## 1. Introduction

Brucellosis remains endemic in sub-Saharan Africa, including South Africa (SA), posing a persistent challenge to livestock productivity and public health [1,2]. The disease is particularly prevalent in the Highveld provinces, including Gauteng, the Free State, Mpumalanga, and parts of North-West and Limpopo provinces, where intensive cattle farming practices and historical infection cycles have contributed to its continued presence [3].

The SA bovine brucellosis control scheme, launched in 1979 and reinforced by legislation in the 1980s, prescribed vaccination of heifers (4–8 months) with the S19 vaccine, together with compulsory testing and slaughter of high-risk cattle, such as dairy cattle, cattle for export, or infected livestock. Despite these efforts, outbreaks and an increased prevalence of brucellosis among tested cattle and other livestock have been reported. Historical serological surveys indicate low apparent seroprevalence in some cattle populations in KwaZulu-Natal, with 1.5% at the Cato Ridge abattoir (1981–1982) and 1.45% in rural cattle (2001–2003) [1,4]. These local surveys, together with regional reviews and national control policy documents, support the observation of relatively low prevalence in the sample population during the 1980s to early 2000s, with limited knowledge of national prevalence [1,3,4,5]. Retrospective studies using serological tests on cattle samples submitted to the veterinary laboratory reported a relatively higher prevalence of 6.3% in SA [6], based on bovines tested from all provinces between 2007 and 2015. More recently, data from the Limpopo and Free State provinces indicated a seroprevalence of 4.3% for the period 2013–2022 [7]. The increase in brucellosis prevalence emphasizes the need for strengthened surveillance and disease management strategies in these high-risk regions [8].

According to the SA bovine brucellosis scheme, “new herds” entering the brucellosis programme must undergo two negative brucellosis tests, with the tests conducted no less than two months and not more than five or six months apart, before a CA3 declaration is issued by the State Veterinarian. Thereafter, annual retesting is required to maintain a disease-free status [3]. A retrospective study reporting serological data from cattle over nine years [6] indicated that 691,529 animals were tested. Compared to the total cattle population of 14 million in SA [9], this corresponds to an annual testing coverage of less than 1%, indicating that the majority of cattle in SA are not routinely sampled outside of outbreak investigations or targeted surveillance programmes. In endemic regions, limited routine testing and inadequate population coverage may contribute to underestimation of the true prevalence, as chronically or latently infected animals can test seronegative. Latently infected animals are defined as those in the early incubation stage, infected but without detectable antibodies, whereas chronically infected animals harbour localized infections (typically in the udder, uterus, or lymph nodes) with waning or fluctuating antibody titers below test detection limits, and may shed *Brucella* intermittently [10,11,12].

In SA, brucellosis diagnosis and control rely primarily on serological tests, particularly the rose Bengal test (RBT) and complement fixation test (CFT), conducted by accredited laboratories under the bovine brucellosis scheme. The RBT is widely used as a screening test due to its rapidity, simplicity, affordability, and high sensitivity [13,14,15]. Positive RBT results are confirmed by CFT, which provides highly specific detection of IgG antibodies [10,11,12]. However, CFT is technically demanding, costly, difficult to interpret, and challenging to standardize [16,17,18,19]. In endemic areas such as SA, the use of serological tests such as RBT and CFT, detecting mainly IgM and IgG1, may fail to identify latent or chronic infections due to reduced sensitivity [10,11,12,20]. The iELISA, which detects a broader range of IgG, including IgG2, exhibits higher sensitivity for chronic infections [11,21,22,23,24]. However, as with other serological tests, careful interpretation of the results is required, particularly in vaccinated animals, due to the potential for vaccine-induced false positives. Thus, while the standard RBT and CFT combination may miss chronically infected carriers, using iELISA can improve detection of low-level antibody responses. Because RBT and CFT may fail to detect all chronic and latent infections, integrating an indirect enzyme-linked immunosorbent assay (iELISA) may improve diagnostic accuracy [25,26]. This underscores the need for enhanced surveillance strategies to more effectively monitor and control brucellosis at the national level.

PCR assays further enhance diagnostic capability by detecting *Brucella* DNA in tissue and blood, allowing identification of chronic and latent infections that serology may miss [27]. Common PCR targets include 16-23S rDNA interspacer (ITS), *bcsp*31, species-specific *IS*711 regions used in AMOS-PCR and real-time PCR assays [26,28,29,30,31]. Among the terrestrial *Brucella* species, *B. abortus* and *B. melitensis* are predominant in livestock, particularly cattle, goats, and sheep [3]. *Brucella ovis* is enzootic in several provinces with intensive sheep farming [32]. Although *B. suis* is an important cause of reproductive losses in pig industries globally, it has not been reported in SA to date [3].

Active surveillance, vaccination, traceability, and reporting in SA remain limited [3,33]. In the absence of active surveillance, abattoirs serve as valuable points for passive monitoring of brucellosis, particularly in subclinical animals. Animals slaughtered at abattoirs undergo pre- and post-mortem examination, and any lesions are reported to the authorities [34]. Additionally, abattoirs provide access to tissues such as the liver, lymph nodes, spleen, lung, and reproductive organs for culture and PCR testing, enabling laboratory confirmation of infections. The study aimed to assess the seropositivity of livestock slaughtered at abattoirs in Limpopo and the Free State using RBT and CFT as primary tests, and to compare the diagnostic outcomes with those of iELISA and PCR to improve diagnostic accuracy. Data generated from this study will contribute to understanding the disease burden in these provinces and inform the enhancement of control strategies.

## 2. Materials and Methods

### 2.1. Study Area and Design

A cross-sectional study was conducted to determine the seropositivity of brucellosis and to isolate and characterize *Brucella* spp. from cattle and sheep slaughtered at various abattoirs across the Limpopo and Free State provinces of SA. From each province, 290 animals, which included 192 cattle and 98 sheep, were samples from 2023 to 2024, with blood, liver, lung, spleen, and lymph nodes sampled from each animal.

### 2.2. Sample Size Determination

A systematic simple random sampling method was used to determine the sample size and statistical power. The sample size was estimated using Epi-Info version 7, assuming an expected prevalence of 50% for cattle and 15% for sheep, with a 5% margin of error, a design effect of 1, and a single cluster. Because the precise baseline prevalence in the specific areas of interest was unknown, expected prevalence of 50% for cattle and 15% for sheep was assumed, with the latter based on the generally low prevalence of brucellosis in South African small ruminants. At a 95.0% confidence level, the calculated sample sizes were 384 cattle and 196 sheep. Demographic information, including species, sex, and age, was recorded for each animal. Due to the convenience sampling approach employed in this study, only small ruminants aged 12–24 months were available for slaughter during the sampling visits. The age categories used in the study were therefore grouped to facilitate statistical analysis and interpretation of the data. The samples were transported to the laboratory for processing on a cold chain within 24 h, observing all biosecurity and Animal Diseases Act (Act 35 of 1984) protocols, and national regulations for the transportation of biohazardous materials (Act 93 of 1996).

### 2.3. Sample Collection

Blood samples were collected at exsanguination, following stunning, into 5 mL gel clot activator Vacutainer tubes. The blood was allowed to clot and centrifuged at 3000 rpm for 10–15 min. After centrifugation, the serum was aliquoted into 2 mL centrifuge tubes and stored at −80 °C until tested. Tissue samples corresponding to the blood samples of each animal were collected. These included samples from lymph nodes, spleen, lung, and liver, which were collected from each animal because these organs are part of the reticuloendothelial (mononuclear phagocyte) system, where *Brucella* organisms preferentially localize and multiply. The lymph nodes, including retropharyngeal, parotid, submandibular, and mesenteric from the same animal, were pooled into a single composite sample as included in previous studies [35,36]. All tissue samples were collected aseptically using sterile instruments and placed in sterile 50 mL containers. Samples were transported under cold chain conditions to the laboratory for further microbiological and molecular analyses.

### 2.4. Brucellosis Positivity Analysis

#### 2.4.1. Serological Methods

##### Rose Bengal Test (RBT)

The RBT was conducted following the procedure described by the World Organisation for Animal Health (WOAH, formerly OIE, 2009). A total of 580 serum samples were screened for the presence of *Brucella* antibodies. Briefly, equal volumes (25 μL) of serum sample and rose Bengal antigen (Onderstepoort Biological Products (OBP), Pretoria, South Africa) were added into a clean Rose Bengal test plate (Thermofisher Scientific, Johannesburg, South Africa). The antigen and serum were thoroughly mixed and gently shaken on an orbital shaker (Heidolph, Schwabach, Germany) for four minutes at room temperature. Following the shaking, the plates were examined under an illuminated light box (Medicare, Johannesburg, South Africa) for visible agglutination. Samples showing distinct agglutination were recorded as positive, while those without agglutination were considered negative. Reference positive and negative sera (*B. abortus*; OBP, Pretoria, South Africa) were used as controls in each run. All sera that tested RBT positive were subsequently subjected to CFT for confirmatory diagnosis.

##### Complement Fixation Test (CFT)

To confirm the *Brucella*-positive samples identified by the RBT, the CFT was performed as a confirmatory assay. *Brucella ovis* infection cannot be detected using the RBT; therefore, all sheep serum samples were additionally tested using *B. ovis* antigen (Middleburg Provincial Veterinary Laboratory, Eastern Cape, South Africa), while RBT-positive sera were tested using *B. abortus* antigen (Onderstepoort Biological Products, Pretoria, South Africa). The CFT was performed at the Agricultural Research Council, Onderstepoort Veterinary Research (ARC-OVR) laboratory, Pretoria, South Africa, following WOAH, formerly OIE standard protocol [37].

The endpoint table was used as a guideline to read the positive and negative test samples. Test results of ≥30 IU/mL were considered positive for both assays when vaccination history was unknown. When vaccination history was known, a higher cut-off value of ≥60 IU/mL was applied.

##### Indirect Enzyme-Linked Immunosorbent Assay (iELISA)

For the detection of *Brucella* antibodies in serum, a multispecies brucellosis kit (Innovative Diagnostics, Grabels, France) was used according to the manufacturer’s recommendation. According to the manufacturer’s instructions, an S/P reading < 110 is considered negative, S/P > 110 and <120 is doubtful, and S/P> or =120 is concluded as a positive result.

##### Serological Criteria for Brucellosis-Positive Animals

The current diagnostic criteria used in the SA bovine brucellosis control scheme involve screening with RBT, followed by confirmation of positive samples using CFT. In this study, an animal was classified as ‘infected’ upon laboratory confirmation of *Brucella* spp. based on bacterial isolation and/or AMOS-PCR detection, as well as a series of serological tests (any two or more of RBT, CFT, and iELISA) results.

#### 2.4.2. Molecular Detection of Brucella from Tissues

##### DNA Extraction

DNA was extracted using the PureLink genomic DNA extraction kit (Thermofisher Scientific, Johannesburg, South Africa) according to the manufacturer’s recommendations. Tissue samples were finely cut and placed into 2 mL microcentrifuge tubes (Thermofisher Scientific, Johannesburg, South Africa). Each sample was homogenized with 180 µL of digestion buffer and 20 µL of proteinase K, followed by overnight incubation on a heat block at 55 °C to ensure complete cell lysis. Following incubation, 200 µL of the lysate was transferred to a clean 2 mL microcentrifuge tube, and 20 µL of RNase enzyme was added to degrade any RNA. The mixture was incubated at room temperature and briefly vortexed. Subsequently, 200 µL of DNA binding buffer was added, mixed by vortexing, followed by the addition of 200 µL of absolute alcohol and mixing by vortexing. A total of 620 µL of the prepared mixture was loaded onto a DNA spin column and centrifuged at 10,000 rpm for 1 min. The column was then washed with 500 μL of wash buffer 1 and centrifuged for 1 min at 10,000 rpm. This step was followed by washing with 500 µL of wash buffer 2 at 15,000 rpm for 3 min. DNA was eluted from the column using 100 µL of elution buffer at 10,000 rpm for 1 min. The DNA concentration and purity were assessed with a spectrophotometer at 260 nm. Extracted DNA was finally stored at −80 °C until use.

##### Detection of the *Brucella* Genus

To detect bacteria belonging to the *Brucella* genus, a genus-specific simplex PCR assay targeting the 16S-23S rDNA interspacer region (ITS) amplifying a 214 bp fragment was performed as described by [28]. Extracted DNA from the lymph nodes, spleen, lung, and liver tissues of each animal (n = 2320 from 580 animals) was analyzed for *Brucella* DNA using the ITS primers. In brief, a 12 μL PCR reaction mix was prepared, comprising 6.5 µL Red Taq Mastermix (1×; Thermofisher Scientific, Johannesburg, South Africa), 0.3 µL (0.2 µM) forward primer, 0.3 µL reverse primer (0.2 µM), and 4.9 µL of nuclease-free water. For each reaction, 3 µL of DNA was added to a final volume of 15 µL. Amplification was performed using a Veriti thermal cycler (Thermofisher Scientific, Waltham, MA, USA) with a heated lid, preheated to 105 °C. The PCR cycling conditions consisted of 95 °C for 3 min, followed by 35 cycles of 95 °C for 1 min, 60 °C for 2 min, 72 °C for 2 min, and a final extension of 72 °C for 5 min. The expected PCR product of 214 bp was visualized by electrophoresis on a 2% agarose gel (Lasec SA (Pty) Ltd., Midrand, South Africa) stained with ethidium bromide (0.03 µL/mL). Electrophoresis was performed at 120 V for 1 h, and DNA bands were visualized under UV illumination using a Bio-Rad ChemiDoc™ XRS molecular imaging system (Bio-Rad, Hercules, CA, USA). *B. abortus* bv. 1 BCCN R4 DNA (OBP, South Africa) was used as a positive control.

##### Detection of *Brucella* Species

For the detection and differentiation of *Brucella* species, an AMOS (detecting *B. abortus* bv 1, 2, and 4, *B. melitensis* bv 1–3, *B. ovis*, *B. suis* bv 1) multiplex PCR assay was conducted as described by [38,39,40] with minor modifications. In brief, the assay used species-specific primers targeting *B. abortus* (498 bp), *B. melitensis* (976 bp), *B. ovis* (731 bp), *B. suis* (285 bp), and reverse primer (IS711) [36]. Each reaction contained four species-specific forward primers at a final concentration of 0.1 µM and 0.2 µM reverse primer IS711. PCR cycling conditions consisted of an initial denaturation at 95 °C for 5 min, followed by 95 °C for 1 min, 35 cycles of 55.5 °C for 2 min, 72 °C for 2 min, and a final extension step at 72 °C for 10 min. Specific amplicon sizes were determined using agarose electrophoresis. *B. abortus* bv. 1 BCCN R4 DNA and *B. melitensis* Rev. 1 DNA, *B. ovis* strain RC48 DNA, and *B. suis* DNA were used as positive controls. Due to the low concentration of our DNA samples, the Bruce-ladder PCR assay [41] could not be performed. In this study, an animal was considered PCR positive for brucellosis when the sample yielded a positive amplification in the genus-specific ITS-PCR assay. AMOS PCR was then used to identify the *Brucella* species in infected animals.

### 2.5. Conventional Microbiological Techniques

#### Bacterial Isolation

All PCR and seropositive samples were subjected to culture. Therefore, tissue samples from the liver, lung, spleen, and lymph node were aseptically cut and placed into 2.5 mL cryovials, emulsified with deionized water, and macerated with a homogenizer. Following homogenization, 200 µL of the homogenate was plated onto Farrell and blood agar and incubated at 37 °C in 5–10% CO_2_ and discarded if no colonies were observed after 15 days. All procedures were performed under biosafety level-2 plus (BSL-2+) following the standard laboratory protocols. The culture isolation of *Brucella* spp. is the gold standard for the diagnosis of brucellosis. Therefore, any animal from which the *Brucella* species is successfully isolated is classified as a confirmed positive case or animal.

### 2.6. Ethical Consideration

Ethical approval to conduct this study was obtained from the University of Pretoria, Animal Ethics Committee (REC 073-23). The Section 20 approval for animal disease research under the Animal Disease Act (Act No. 35 of 1984) was obtained from the Directorate of Animal Health, Department of Agriculture, Land Reform and Rural Development (DALRRD) for both bovine and ovine samples.

### 2.7. Data Analysis

Descriptive statistical analyses were performed to ascertain the number and proportion of *Brucella-*positive samples, stratified by diagnostic tests, geographic location, animal species, gender, and age group, with corresponding 95% confidence intervals (CIs). The Chi-squared test or Fisher’s exact test, where appropriate, was used to determine the association between *Brucella* positivity and categorical variables, with a *p*-value considered statistically significant. The level of agreement between diagnostic tests was evaluated using Cohen’s kappa (κ) statistic, as described by Landis and Koch (1977) [42]. The kappa result was interpreted as follows: kappa ≤ 0 = no agreement; 0.01–0.20 = none to slight; 0.41–0.60 = moderate; 0.61–0.80 = substantial; and 0.81–1.00 = almost perfect agreement. The information was captured in an Excel spreadsheet and statistically analysed using IBM SPSS v.29.

## 3. Results

### 3.1. Demographical Analysis

A total of 580 animals were tested, comprising 384 cattle and 196 sheep, with equal numbers of cattle (n = 192) and sheep (n = 98) sampled across both Limpopo and the Free State. Among cattle from Limpopo, females and males accounted for 44.8% (n = 86) and 55.2% (n = 106), respectively, whereas cattle from the Free State were predominantly female, with 75.5% (n =145) females and 24.5% (n = 47) males. Among sheep sampled in Limpopo, females accounted for 15.3% (n = 15) and males for 85.0% (n = 83), while sheep from the Free State comprised 74.5% (n = 73) females and 25.5% (n = 25) males. Regarding age distribution, most cattle from Limpopo were 1–2 years (72%; n = 140), followed by those older than 3 years (24.5%; n = 47), with only 2.6% (n = 5) aged 2–3 years. In contrast, cattle from the Free State were predominantly aged 2–3 years (59.4%; n = 114), followed by those older than 3 years (39.58%; n = 76), with only two (1.0%) animals aged 1–2 years. All sheep sampled from both provinces were between 1 and 2 years of age.

### 3.2. Positivity of Brucellosis

Of the bovines sampled in the Free State (n = 192), 2.1% (4/192) tested positive for RBT-CFT (F (female):M (male) = 1:1), and 5.2% (10/192) for RBT-iELISA (F:M = 4:1), all aged 2–3 years. The RBT detected 5.2% (10/192) bovine positives, all of which were confirmed by iELISA, while iELISA detected 9.4% (18/192), with 5.7% (11/192) detected by RBT, PCR, and/or CFT. The bovines that were only iELISA-positive accounted for 3.7% (8/192; 2.0–7.7) and were classified as suspect, with all animals older than 2 years (Table 1). ITS-PCR detected 14.1% (27/192) positives (Supplementary Tables S1 and S2), of which all were seronegative except for one that was ELISA-positive, with 77.7% (21/27) females, most being older than 3 years. AMOS-PCR identified 88.9% *B. abortus* mainly (24/27) and *B. melitensis* in 2/27 (7.4%). None of the ovines were seropositive, but *Brucella* DNA was also detected in 26.5% (26/98), comprising 11.5% (3/26) males and 88.5% females, all being 1–2 years of age (Table 2). AMOS-PCR identified *B. abortus* to be predominant, at 80.8% (21/26), in animals aged 1–2 years (Table 2).

Brucellosis DNA detection by ITS PCR in the Free State was lower in cattle (14.1%; 27/192) than in sheep (26.5%; 26/98), indicating a higher burden in ovine populations (Supplementary Tables S1 and S2). In both species, *B. abortus* predominated (88.9% in cattle; 80.8% in sheep), with smaller contributions from *B. melitensis* and occasional mixed infections. PCR positivity in cattle was slightly higher in females than in males and increased markedly with age, and it was the highest in animals older than 3 years (31.6%). In contrast, ovine positivity was higher in females (Table 1). Across tissues, the spleen and liver yielded the highest *Brucella* DNA detection, with PCR detection rates in both species (Supplementary Tables S1 and S2). Concordance between serology and PCR was poor, with most seropositive animals testing negative by PCR.

Bovines in Limpopo (n = 192) were 3.1% (6/192) RBT-CFT, as well as iELISA-CFT-positive, of which 2 were PCR-positive, with most being older than 3 years of age (F:M ratio = 5:1), except one that was also PCR-positive, being 1–2 years old (Table 1 and Table 2, Supplementary Table S2). The RBT-iELISA positives included 4.2% (8/192) (F:M 7:1), with most being older than 3 years, except for two that were 1–2 years old (Table 1). The RBT positives consisted of 8/192, all of which were iELISA-positive, while iELISA positives included 13/192, of which 10/192 were detected by RBT, CFT, and/ or PCR (Table 1). The bovines that were only ELISA-positive were 2.6% (5/192), with 3 suspects and 2 confirmed by PCR, with all animals older than 2 years, except for a single PCR positive (1–2 years). PCR positives bovines constituted 22.9% (44/192) (F:M ratio = 1:1) from Limpopo, mainly comprising 1–2-year-old animals, with 88.6% (39/44) being seronegative. *Brucella abortus* was detected in 65.9% (29/44) (F:M = 1:1.4) and 34.1% (15/44) *B. melitensis* (F:M = 2:1) (Supplementary Table S2). All ovines tested seronegative, but *Brucella* DNA was detected in 30.6% (30/98), all aged 1–2 years (F:M ratio = 1:4), with *B. abortus* detected in 83.3% (25/30) (F:M ratio = 1:4) and *B. melitensis* in 13.3% sheep (Table 1 and Table 2). Thus, brucellosis DNA was detected by ITS PCR in 22.9% (44/192) of bovine and 30.6% (30/98) of ovine tissue samples in Limpopo, indicating a higher burden in cattle populations. In both species, *B. abortus* predominated, accounting for 65.9% of positive cattle and 83.3% of positive sheep, while *B*. *melitensis* was also present, particularly in cattle (34.1%), with only one mixed infection detected in sheep (Table 2, Supplementary Table S2).

In both provinces, all bovines tested (n = 384) were 2.60% (10/384) brucellosis RBT-CFT, as well as 2 ELISA positives and 2 PCR positives, 4.7% (18/384) RBT-iELISA, and 18.5% (71/384) PCR positives. The bovines that were only ELISA-positive were 4.2% (8/192) and are thus suspect. In tested bovines that are brucellosis infected, using the criteria of any two serological tests, consisting of 5.2% (20/384), or serology tests and/or PCR positives, 22.92% (88/192).

### 3.3. Detection of Brucellosis by PCR

A total of 2320 DNA extracts (liver, lung, spleen, lymph nodes) obtained from 580 animals were screened using an ITS (16S-23S rDNA interspace region) genus-specific PCR assay for the detection of the *Brucella* genus. The assay detected *Brucella* DNA in 8.2% (n = 191) of samples, corresponding to 21.9% (n = 127) of tested animals. Of the 127 positive animals, 27 bovines (F:M; 3.5:1) and 26 ovines (F:M = 7.7:1) originated from the Free State, with *B. abortus* dominating in females of both species, while *B. melitensis* was predominant in males (Supplementary Tables S1 and S2). In Limpopo, 44 bovines (F:M = 1:1) and 30 ovines (F:M = 1:4) were positive. *Brucella abortus* was predominant in male bovines and female ovines, while *B. melitensis* was predominant in females of both species (Table 2, Supplementary Table S2). Stratification by sample type showed the highest detection rate in the liver and spleen across all species and provinces. Seropositive animals showed relatively low concordance with PCR detection, particularly in cattle, where most seropositive animals were PCR-negative.

### 3.4. Detection of Brucellosis by Culture

Tissues from animals that were positive by RBT and iELISA, and positive by CFT and PCR, were homogenized in 200 µL sterile deionized water and cultured on Farrell and blood agar. All samples tested were negative for the *Brucella* species after 15 days of incubation. Therefore, the proportion of *Brucella* isolation from culture was 0%.

### 3.5. Data Analysis

A substantial agreement (Kappa = 0.65; *p* < 0.001) was observed between RBT and iELISA assays, with 18 reactive samples by both tests. All 18 positive samples were of bovine origin. All RBT-CFT-positive samples were also positive by iELISA; however, an additional 13 samples were positive by iELISA alone. Significant associations (*p* < 0.001) were observed between RBT-iELISA results and species, sex, and age. Similarly, RBT-CFT and RBT-iELISA results showed significant associations with species, sex, and age. In contrast, no statistically significant associations were observed between RBT-PCR, iELISA-PCR, or CFT-PCR results and species, sex, and age. Additionally, no agreement (Kappa < 0) was observed between serological methods and PCR.

## 4. Discussion

The findings of this study confirm that brucellosis remains endemic in SA, with detectable infection in both cattle and sheep in the Limpopo and Free State provinces. Overall seropositivity using conventional RBT-CFT testing was low, ranging from 2.1 to 3.1% in cattle across the two provinces, while iELISA detected additional suspect animals, increasing the apparent prevalence to 4–9%. In contrast, PCR assays revealed substantially higher positivity, with 14.1% in Free State and 22.9% in Limpopo cattle, with most PCR-positive animals testing seronegative, indicating the presence of latent or chronic infections undetectable by routine serology. A similar pattern was observed in ovines, with PCR positivity (26–30%) far exceeding serological detection, and *B. abortus* predominating in both species. These results highlight the limitations of relying solely on RBT and CFT for estimation, particularly in endemic regions where chronic and latent infections are common. The discrepancy between serology and PCR underscores the need to integrate more sensitive diagnostics, such as iELISA and PCR, to accurately capture the true burden of brucellosis. Furthermore, the low annual coverage of cattle testing under the national brucellosis scheme (<1% per year) suggests that most animals are not routinely monitored outside of targeted surveillance or outbreak investigations, emphasizing gaps in the current control measures and the critical need for enhanced, province-specific surveillance strategies.

In both provinces, seropositivity was confined to older cattle, reflecting cumulative exposure over time, with an even sex distribution in the Free State but a higher positivity in females in Limpopo. In the Free State, PCR positivity in cattle increased with age, with the highest observed in animals older than three years, supporting prolonged exposure and potential persistence of infection [43]. These findings are consistent with reports from South Sudan, Zambia, Kenya, and Ethiopia, which also identified higher seropositivity in older female animals [31,44,45,46]. The higher positivity in older animals may be attributed to herd management practices, where females are retained longer for breeding, thereby increasing the duration of exposure [45,47,48]. In addition, sexually mature and pregnant animals are generally considered more susceptible to infection than sexually immature animals, likely due to factors such as the presence of erythritol in reproductive organs, which can promote *Brucella* replication [41,49,50,51]. However, reproductive organs were not included in the current study, and therefore, this aspect could not be directly addressed within the context of the sampled tissues.

In contrast, Ref. [34] reported higher seropositivity in animals younger than 2 years, highlighting potential variability in transmission dynamics across production systems. Similarly, in Limpopo, most PCR-positive animals (female cattle and sheep) were 1–2 years old, suggesting early-life exposure, potentially in utero or via milk [43]. This finding is of concern as these animals may remain carriers and contribute to future outbreaks, particularly during their first pregnancies [11]. The higher detection in females is consistent with the role of reproductive tissues as primary sites of *Brucella* localization and shedding [52].

Species-specific identification revealed that *B. abortus* was the predominant pathogen in both cattle (65–88% of positives) and sheep (80–83%), whereas *B. melitensis* was detected primarily in cattle (34%), with occasional mixed infections. These results are in agreement with previous reports from SA [1,35,53,54], confirming the continued dominance of *B. abortus* in livestock populations. However, detection of *B. melitensis* in cattle suggests cross-species transmission, particularly in mixed farming systems [55]. These findings indicate that, although cattle remain the primary reservoir of brucellosis in SA, small ruminants may contribute to infection dynamics through spill-over events [56]. This “spillover” from small ruminants to cattle underscores the need for integrated, multi-species surveillance and control strategies aligned with a One Health approach [57,58,59], rather than a sole focus on bovine populations. Furthermore, the observed age- and sex-associated patterns of infection further suggest that reproductive management, targeted vaccination, and focused monitoring of older and female animals are critical for reducing disease persistence and transmission.

While the standard RBT-CFT algorithm identified only 2.6% of cattle as positive, ITS-PCR detected *Brucella* DNA in 18.5% of the same population. The fact that 97% of PCR-positive cattle were seronegative suggests a high level of subclinical, chronic carriage that current national protocols are failing to capture. This finding is consistent with reports by [26,50,60], which also identified PCR-positive but seronegative samples, suggesting that some infected animals may not be detected by traditional tests alone, particularly in endemic regions. In such areas, standard serological protocols, especially when not applied through regular, systematic testing, will fail to detect latent and chronically infected animals.

Despite being entirely seronegative across all conventional tests, sheep exhibited a higher PCR positivity rate (28.6%) than cattle (18.5%). These findings are consistent with reports from East Africa. For instance, Ref. [61] reported that sheep in South Sudan remained seronegative despite the presence of the disease in the region. Furthermore, the higher PCR positivity in seronegative animals in this study echoes the findings by [62], where nearly 30% of seronegative sheep were found to harbour *Brucella* DNA, suggesting that sheep often act as silent, undetectable reservoirs in the endemic landscape. This indicates that sheep in the Free State and Limpopo may act as silent reservoirs for the disease, highlighting that reliance on RBT and CFT for small ruminants is clearly insufficient, as these animals may carry the pathogen without a detectable humoral response. This “silent” infection poses a significant risk to public health and trade, as sub-clinically infected animals may enter the food chain without being detected and expose consumers to infection, while also serving as a source of occupational exposure to individuals in close contact with livestock, including farmers, veterinarians, and abattoir workers. Furthermore, they may facilitate cross-species transmission among animals, negatively affecting animal health, welfare, and livestock productivity, particularly in mixed and intensive farming systems common in SA [63,64].

The 0% isolation rate from culture, despite high PCR positivity, highlights the technical challenges of brucellosis diagnosis. The sources suggest this failure is likely due to low bacterial loads in chronic, non-clinical cases; the non-viability of organisms in the tissues of animals that may have been partially controlled by the immune system; and contamination by faster-growing bacteria during the long incubation period required for *Brucella* [12,65]. Furthermore, the cultures in this study were derived from tissues collected from apparently healthy animals at abattoirs, rather than from placenta and/or aborted material, where the bacterial load is typically highest, and cultivation is most successful. This reinforces the argument that PCR should be integrated into diagnostics where tissue or aborted material is available, as it bypasses the sensitivity and biosafety limitations of culture when tissue samples such as those from an abattoir are available.

The current South African bovine brucellosis control scheme (established in 1979) primarily relies on traditional serological diagnostic approaches. In the current study, substantial agreement (Kappa = 0.65) was observed between RBT and iELISA, while iELISA identified an additional 13 samples that were not detected by CFT. These findings suggest that iELISA may have potential utility as a complementary tool in large-scale surveillance programmes, particularly in endemic settings. Furthermore, several countries have progressively incorporated iELISA-based assays into routine brucellosis surveillance due to limitations associated with CFT, including challenges related to standardization, high reagent requirements, reagent availability, ethical considerations, and logistical constraints [66,67]. In contrast, iELISA is easier to perform, more readily standardized and widely supported by existing laboratory infrastructure. The inclusion of iELISA may improve the detection and tracing of exposed animals, particularly in unvaccinated populations.

## 5. Conclusions

The study demonstrated that *Brucella* is prevalent in the Limpopo and Free State provinces, with confirmed detection in cattle and sheep slaughtered at selected abattoirs. These findings highlight the value of passive abattoir-based surveillance in providing critical insight into the occurrence and distribution of *Brucella* within livestock populations. The presence of this pathogen at the slaughter level underscores a significant risk to food safety and public health, while also posing potential constraints on livestock productivity and trade. Therefore, there is a need for strengthened national control and prevention strategies to mitigate the impact of brucellosis. In particular, the incorporation of more sensitive and specific diagnostic tools, such as iELISA as serological tests and PCR, may be considered in routine surveillance and testing programmes to enhance early detection and improve disease management in SA.

## 6. Limitations of the Study

The study detected *Brucella* antibodies and DNA in slaughtered cattle. The findings should be interpreted within the limitations of the study design. The samples represented a statistically selected subset of the slaughtered animals rather than a comprehensive surveillance programme encompassing all slaughtered or live populations. Therefore, the results primarily support the presence of *Brucella* infection in animals slaughtered at selected abattoirs in the two provinces, but do not allow definitive epidemiological inferences regarding the true burden or distribution of the disease. Under these conditions, it was not possible to comprehensively assess the diagnostic sensitivity of the assays for disease control. The data collected from the abattoir did not include the herd information and vaccination history of the animals, which makes it difficult to establish whether the animals were vaccinated. Additionally, the absence of data on animal breed limits the ability to evaluate breed-associated susceptibility or risk factors. The sample size used in this study does not give a clear representation of the animal population in the two provinces. Furthermore, the DNA concentrations extracted from tissues were insufficient for conducting Bruce-ladder PCR, limiting further characterization. The unavailability of certain tissues, including sub-mammary lymph nodes, mammary gland, uterus, and testicles, during slaughterhouse sampling may have reduced the sensitivity of the culture method. The failure to recover viable isolates prevented the application of downstream analyses such as whole genome sequencing, which could have provided deeper insight into strain diversity.

## Figures and Tables

**Table 1:** Seropositivity of brucellosis by diagnostic test, including rose Bengal test (RBT), complement fixation test (CFT), indirect ELISA (iELISA), and ITS-PCR by host species and province.

	Category	Animal Tested	RBT (%) 95% CI	*p*- Value	iELISA (%) 95% CI		RBT, CFT (%) 95% CI	*p*- Value	RBT, iELISA (%) 95% CI	*p*- Value	PCR (%), 95% CI	
Free Sate												
Species	Bovine	192	10 (5.2) 2.7–9.0	0.21	18 (9.4) 5.9–14.1	0.02	4 (2.1) 0.7–4.9	<0.001	10 (5.2) 2.7–9.0	<0.001	27 (14.1) 9.7–19.5	0.009
	Ovine	98	0 (0) 0.0–2.5		0 (0.0) 0.0–2.5		0 (0) 0.0–2.5		0 (0.0) 0.0–2.5		26 (26.5) 18.6–35.9	
Gender	Female	218	8 (3.7)	0.70	15 (6.9)	0.41	2 (0.9)	<0.001	8 (3.7)	<0.001	44 (20.2)	0.14
			1.7–6.8		4.1–10.8		0.2–2.9		1.7–6.8		15.3-25.9	
	Male	72	2 (2.8)		3 (4.2)		2 (2.8)		2 (2.8)		9 (12.5)	
			0.6–8.6		1.2–10.7		0.6-8.6		0.6–8.6		6.4–21.6	
Age	1–2 yrs	100	0 (0.0)	0.03	0 (0.0)	0.003	0 (0.0)	<0.001	0 (0.0)	<0.001	27 (27.0)	<0.001
			0.0–2.5		0.0–2.5		0.0–2.5		0.0–2.5		19.0–36.3	
	2–3 yrs	114	9 (7.9)		13 (11.4)		4 (3.5)		9 (7.9)		2 (1.8)	
			4.0–13.9		6.5–18.2		1.2–8.1		4.0–13.9		0.4–5.5	
	>3 yrs	76	1 (1.3)		5 (6.6)		0 (0)		1 (1.3)		24 (31.6)	
			0.1–6.0		2.6–13.8		0.0–32		0.1–6.0		22.0–42.6	
Limpopo												
Species	Bovine	192	8 (4.2)	0.70	13 (6.8)	0.008	6 (3.1)	<0.001	8 (4.2)	<0.001	44 (22.9)	0.16
			2.0–7.7		3.8–11.0		1.3–6.3		2.0–7.7		1.7–2.9	
	Ovine	98	5 (5.1)		0 (0.0)		0 (0)		0 (0.0)		30 (30.6)	
			2.0–10.8		0.0–2.5		0.0–2.5		0,0–2.5		22.2–40.2	
Gender	Female	101	7 (6.9)	0.10	12 (11.9)	<0.001	5 (5.0)	<0.001	7 (6.9)	<0.001	28 (72.3)	0.53
			3.2–13.1		6.7–19.2		1.9–10.5		3.2–13.1		19.7–37.0	
	Male	189	6 (3.2)		1 (0.5)		1 (0.5)		1 (0.5)		46 (24.3)	
			1.3–6.4		0.1–2.4				0.1–2.4		18.6–30.8	
Age	1–2 yrs	238	7 (2.9)	0.17	3 (1.3)	<0.001	1 (0.4)	<0.001	2 (0.8)	<0.01	69 (29.0)	<0.001
			1.3–5.7		0.4–3.3		0.0–1.9		0.2–2.7		23.5–35.0	
	2–3 yrs	5	0 (0)		0 (0.0)		0 (0)		0 (0.0)		0 (0.0)	
			0.0–37.9		0.0–37.9		0.0–37.9		0.0–37.9		0.0–37.9	
	>3 yrs	47	6 (12.8)		10 (21.3)		5 (10.6)		6 (12.8)		5 (10.6)	
			5.5–24.4		11.5–34.5		4.2–21.8		5.5–24.4		4.2—21.8	

**Table 2:** Positivity of brucellosis in the Free State and Limpopo provinces by AMOS-PCR directly from tissues.

Species	Province	*B. abortus*	*B. melitensis*	Mixed Infections
Cattle	Free State	88.9% (24/27)	7.4% (2/27)	3.7% (1/27)
	Limpopo	65.9% (29/44)	34.1% (15/44)	0
Sheep	Free State	80.8% (21/26)	15.4% (4/26)	3.8% (1/26)
	Limpopo	83.3% (25/30)	13.3% (4/30)	3.3% (1/30))

## Data Availability

The data that support the findings of this study are available on reasonable request from the corresponding author, E.S.

## Supplementary Materials

The following supporting information can be downloaded at https://www.mdpi.com/article/10.3390/microorganisms14061215/s1, Table S1: Positivity of brucellosis in the Free State by ITS and AMOS PCR directly from tissues; Table S2: Positivity of brucellosis in Limpopo by ITS and AMOS PCR directly from tissues.
