# Supplementary material for: Brucellosis Abattoir Surveillance Using Serology and Molecular Tests Among Livestock in Free State and Limpopo Provinces, South Africa
Source: Microorganisms. 2026 May 27;14(6):1215. doi: 10.3390/microorganisms14061215 (PMC13303647; doi:10.3390/microorganisms14061215)
Supplement: Supplementary file 1 [file microorganisms-14-01215-s001.zip › microorganisms-4281840-supplementary.pdf]

Supplementary Table S1: Positivity of brucellosis in Free State by ITS and AMOS PCR directly from tissues

**Category**	**Animals tested**	**ITS Positive Animals (%)**	***B. abortus* (%)**	***B. melitensis* (%)**	**Mixed infection (%)**	**Category**	**Animals Tested**	**ITS Positive Animals (%)**	***B. abortus* (%)**	***B. melitensis* (%)**	**Mixed infection (%)**
**Species**						**Species**					
Bovine	192	27 (14.1)	24 (88.9)	2 (7.4)	1 (3.7)	Ovine	98	26 (26.5)	21 (80.8)	4 (15.4)	1 (3.8)
**Gender**						**Gender**					
Male	47	6 (12.8)	5 (83.3)	1 (16.7)	0 (0.0)	Male	25	3 (12.0)	2 (66.7)	1 (33.3)	0 (0.0)
Female	145	21 (14.5)	19 (90.5)	1 (4.8)	1 (4.8)	Female	73	23 (31.5)	19 (82.6)	3 (13.0)	1 (4.3)
**Age**						**Age**					
1-2 years	2	1 (50.0)	1 (100)	0 (0.0)	0 (0.0)	1-2 years	98	26 (26.5)	21 (80.8)	4 (15.4)	1 (3.8)
2-3 years	114	2 (1.8)	2 (100)	0 (0.0)	0 (0.0)	2-3 years	0	0 (0.0)	0 (0.0)	0 (0.0)	0 (0.0)
> 3 years	76	24 (31.6)	21 (87.5)	2 (8.3)	1 (4.2)	> 3 years	0	0 (0.0)	0 (0.0)	0 (0.0)	0 (0.0)
**Sample type**						**Sample type**					
Liver	192	12 (6.25)	11 (91.6)	2 (6.5)	0 (0.0)	Liver	98	20 (20.4)	18 (90.0)	1 (5.0)	1 (5.0)
Lung	192	9 (4.7)	7 (77.8)	2 (22.2)	0 (0.0)	Lung	98	4 (4.1)	2 (50.0)	2 (50.0)	0 (0.0)
Spleen	192	16 (8.3)	13 (81.3)	2 (12.5)	1 (6.3)	Spleen	98	8 (8.2)	7 (87.5)	1 (12.5)	0 (0.0)
Lymph nodes	192	8 (4.2)	5 (62.5)	2 (25.0)	1 (12.5)	Lymph nodes	98	5 (5.1)	4 (80.0)	1 (20.0)	0 (0.0)
**Test**						**Test**					
RBT, CFT	4	0 (0.0)	0 (0.0)	0 (0.0)	0 (0.0)	RBT, CFT	0	0 (0.0)	0 (0.0)	0 (0.0)	0 (0.0)
RBT-iELISA	10	0 (0.0)	0 (0.0)	0 (0.0)	0 (0.0)	RBT-iELISA	0	0 (0.0)	0 (0.0)	0 (0.0)	0 (0.0)
RBT only	5	1 (20.0)	1 (100.0)	0 (0.0)	0 (0.0)	RBT only	0	0 (0.0)	0 (0.0)	0 (0.0)	0 (0.0)
iELISA only	8	1 (12.5)	1 (100.0)	1 (0.0)	0 (0.0)	iELISA only	0	0 (0.0)	0 (0.0)	0 (0.0)	0 (0.0)

Supplementary Table S2: Positivity of brucellosis in Limpopo by ITS and AMOS PCR directly from tissues

Category	**Animals tested**	**ITS Positive Animals (%)**	***B. abortus* (%)**	***B. melitensis* (%)**	**Mixed infection (%)**
**Species**					
Bovine	192	44 (22.9)	29 (65.9)	15 (34.1)	0 (0.0)
**Gender**					
Male	106	22 (20.8)	17 (77.3)	5 (22.7)	0 (0.0)
Female	86	22 (25.6)	12 (54.5)	10 (45.5)	0 (0.0)
**Age**					
1-2 years	140	39 (27.9)	26 (66.7)	13 (33.3)	0 (0.0)
2-3 years	5	0 (0.0)	0 (0.0)	0 (0.0)	0 (0.0)
> 3 years	47	5 (11.4)	3 (60.0)	2 (40.0)	0 (0.0)
**Sample type**					
Liver	192	22 (11.5)	16 (72.7)	6 (27.3)	0 (0.0)
Lung	192	12 (6.3)	8 (66.7)	4 (33.3)	0 (0.0)
Spleen	192	18 (9.4)	11 (61.1)	7 (38.9)	0 (0.0)
Lymph nodes	192	13 (0.7)	5 (38.5)	8 (61.5)	0 (0.0)
**Test**					
RBT-CFT	6	2 (33.3)	1 (50.0)	1 (50.0)	0 (0.0)
RBT-iELISA	8	2 (25.0)	1 (50.0)	1 (50.0)	0 (0.0)
RBT only	1	0 (0.0)	0 (0.0)	0 (0.0)	0 (0.0)
iELISA only	5	2 (40.0)	1 (100.0)	1 (0.0)	0 (0.0)

**Category**	**Animals Tested**	**ITS Positive Animals (%)**	***B. abortus* (%)**	***B. melitensis* (%)**	**Mixed infection (%)**
**Species**					
Ovine	98	30 (30.6)	25 (83.3)	4 (13.3)	1 (3.3)
**Gender**					
Male	25	24 (96.0)	20 (80.0)	3 (12.5)	1 (4.2)
Female	73	6 (8.2)	5 (83.3)	1 (1.4)	0 (0.0)
**Age**					
1-2 years	98	30 (30.6)	25 (83.3)	4 (13.3)	1 (3.3)
2-3 years	0	0 (0.0)	0 (0.0)	0 (0.0)	0 (0.0)
> 3 years	0	0 (0.0)	0 (0.0)	0 (0.0)	0 (0.0)
**Sample type**					
Liver	98	20 (20.4)	16 (80)	3 (15.0)	1 (5.0)
Lung	98	10 (10.2)	6 (60.0)	4 (40.0)	0 (0.0)
Spleen	98	10 (10.2)	7 (70.0)	3 (30.0)	0 (0.0)
Lymph nodes	98	4 (4.1)	3 (75.0)	1 (25.0)	0 (0.0)
**Test**					
RBT, CFT	5	0 (0.0)	0 (0.0)	0 (0.0)	0 (0.0)
RBT-iELISA	5	0 (0.0)	0 (0.0)	0 (0.0)	0 (0.0)
RBT only	5	2 (4.0)	2 (100)	0 (0.0)	0 (0.0)
iELISA only	0	0 (0.0)	0 (0.0)	0 (0.0)	0 (0.0)
